# Transcriptome profiling of the fertile parent and sterile hybrid in tea plant flower buds

**DOI:** 10.1186/s41065-019-0090-z

**Published:** 2019-04-18

**Authors:** Linbo Chen, Hao Qu, Lifei Xia, Yue Liu, Huibing Jiang, Yunnan Sun, Mingzhi Liang, Changjun Jiang

**Affiliations:** 1 Tea Research Institute, Yunnan Academy of Agricultural Sciences, Menghai, 666201 China; 2 Yunnan Provincial Key Laboratory of Tea Science, Menghai, 666201 China; 3 State Key Laboratory of Tea Plant Biology and Utilization, Anhui Agricultural University, Hefei, 230036 China

**Keywords:** Tea plant, Sterile floral buds, Differentially expressed genes, Auxin

## Abstract

**Background:**

The tea plant is a crucial economic crop. The floral organ development consumes a large amount of nutrients, which affects the leaf yield. To understand the mechanism by which the tea plant produces sterile floral buds, we obtained a sterile tea plant by artificial hybridization. RNA-sequencing based transcriptome analysis was implemented in three samples to determine the differentially expressed genes (DEGs) related to flower development.

**Results:**

In this study, a total of 1991 DEGs were identified; 1057 genes were up-regulated and 934 genes were down-regulated in sterile hybrid floral buds. These were mainly distributed in the regulation of biological and metabolic processes. Significantly, auxin biosynthesis genes *YUCCA*, *AUX1* and *PIN* were dramatically down-regulated, and *ARF* gene was up-regulated in the sterile hybrid floral buds, and flower development-related genes *AP1*, *AP2* and *SPL* were changed. A total of 12 energy transfer-related genes were significantly decreased. Furthermore, the expression of 11 transcription factor genes was significantly different.

**Conclusion:**

The transcriptome analysis suggested that the production of sterile floral buds is a complex bioprocess, and that low auxin-related gene levels result in the formation of sterile floral buds in the tea plant.

## Introduction

Sterility is a complex phenomenon in plants, the main factors of which include the accumulation of reactive oxygen species, abnormalities in energy metabolism, programmed cell death, imbalance in endogenous hormones and changes in environmental condition [1–3]. In *Arabidopsi*s and cereal grains, floral organ degeneration and abiotic stresses result in sterility. Abortion or degeneration of developing stamens and pistil is the key mechanism used by plants to produce sterile flowers [4].

Among the sterility mechanisms of the plant, flower development is one of the main concerns. It requires early and later organ identity genes. *PINFORMED* (*PIN1)*, *PINOID* (*PID)*, *YUCCA* (*YUC)* and *NAKED PINS IN YUC MUTANT* (*NPY)* are necessary for flower development [5, 6]. Organ identity genes *APETALA1* (*AP1*), *APETALA2* (*AP2*), *APETALA3* (*AP3*), *PISTILLATA* (*PI*) and *AGAMOUS* (*AG*) are essential for the ABC model in *Arabidopsis* [7]. The mutation of these genes causes more petals, fewer stamens, fused floral organs, and valveless gynoecia. In addition, important genes for flower development include *SPL*, *TPD1*, *AMS*, *DYT1*, *SHP*, *WUS* [8–13]. Brassionsteroid and jasmonic acid play a positive role in promoting the formation of stamens and pollen. Gibberellin deficiencies are related to male sterility, and the formation of female flowers requires the presence of ethylene [14–16]. Moreover, flower development is regulated by the coordinated interaction of the transcription factor LEAFY and auxin [17]. However, molecular data are limited for the tea plant; the molecular mechanism of flower development remains unknown.

The tea plant is a crucial cash crop widely distributed around the world. Tea leaves have been used to produce various tea beverages. The floral organ development results in the fall of the yield of tea leaves by consuming a large amount of nutrients. Cultivation of the sterile tea plant is key to increasing the yield. The tea plant is self-incompatible, thus we performed transcriptome sequencing and comparative analysis on three samples, including Foxiang2 (FBH), Fudingbaicha (MBH) and hybrid sterile flowers (ZDH). The aim was to analyze the differentially expressed genes between the fertile and sterile floral buds, and to identify their related bioprocesses and correlative factors. Our results will help to reveal important information on the mechanism of sterility in the tea plant.

## Materials and methods

### Plant materials

The plant materials were five-year-old tea plants (*C. sinensis (L.) O. Kuntze*) from the Tea Research Institute, Yunnan Academy of Agricultural Sciences called ‘Foxiang2’, used as FBH, ‘Fudingbaicha’, used as MBH, and a sterile hybrid, used as ZDH. Flower buds were separately stripped from three tea plants. All of the experiments were performed using three biological replicates. Flower buds were picked on October 16, 2016, after which they were frozen using liquid nitrogen and stored in a freezer at − 80 °C for subsequent mRNA analysis.

### cDNA library construction and sequencing

The construction of cDNA libraries and transcriptome sequencing were completed by Beijing Novogene Technology (Beijing, China). Total RNA was used as the starting sample and was directly added to the 3′-terminal hydroxyl group and the uniquely structured complete phosphoryl groups at the 5′-terminal of the sRNA, followed by reverse transcription to synthesize cDNA. After polymerase chain reaction amplification, polyacrylamide gel electrophoresis was used to separate the target DNA fragments, and the gel was recovered, completing the cDNA library. The effective concentration of the library was > 2 nmol/L, and sequencing was performed using Illumina HiSeq 2000 after the library was certified. All of the experiments were performed using three replicates.

### Transcriptome assembly

The original image data files obtained from the Illumina HiSeq 2000 were subjected to base calling analysis and converted into raw reads. Among the raw reads obtained from the sequencing, the low-quality reads with adaptors were processed to obtain clean reads. The clean reads were assembled separately, and TGICL was used to get the longest non-redundant unigenes. The transcriptome data were deposited to the NCBI SRA database (SRA accession: PRJNA503652).

### Differentially expressed genes (DEGs) test

Differentially expressed genes (DEGs) analysis using DEGseq (three biological replicates per group). DESeq provides statistical routines for determining differential expression in digital gene expression data, using a model based on negative binomial distribution. The resulting *P*-values were adjusted using the Benjamin and Hochberg approach for controlling the false discovery rate. The genes with P-value < 0.05 found by DESeq were differentially expressed.

### GO and KEGG analysis

DEGs were characterized according to Gene Ontology enrichment analysis. GO annotations were provided by the Blast2GO program. Then the GO classification graph was generated by the WEGO.

KEGG was used to analyze the biological process and unigenes annotation of pathway. The results were comparatively analyzed between the KEGG integrated database resource and our data.

### Quantitative real-time PCR assays

Total RNA was isolated using TRlpure reagent (BioTeke, China) according to the manufacturer’s instructions. cDNA was synthesized from total RNA using a PrimeScript RT reagent kit (TaKaRa, Japan). The obtained cDNA was used as a template in SYBR green-based q-PCR (CFX-96, Bio-Rad, Hercules, CA, USA). GAPDH was used for normalization. The primers are shown in Table 1.

**Table 1:** Primer Sequences for q-PCR

Gene name	Primer Sequence (5′ to 3′)
AP2	F:TACAGAGGAGTAACAAGGCATCA
	R:CGTCAAAGTTCGTCACAGCA
JAR1	F:GCTTCCACAACTCAACTCCAGA
	R:CAACAAGGCTCGTGAAATCG
ARF	F:TGAAACAGAGGAGTCAGGCAA
	R:CCAGTCTCATCCCACTCTACCT
IAA7	F:TCCAATGAGAAGAAAGACCCTG
	R:CACCTTCACAAACGCCACA
AUX1	F:ACTGAGGCTGAGGTTGGTGA
	R:TTAGATTTGATGGGCGTGGT
ATL3	F:CACACTAACCCTACCATCAGCA
	R:CAGTGTCTCTGAAACCAGTCCTT
GAPDH	F:GATAGTGTTCACGGTCAATGGA
	R:GCAGCAGCCTTATCCTTATCAG

## Results

### Morphological characteristics of sterile flower buds

**Fig. 1:**
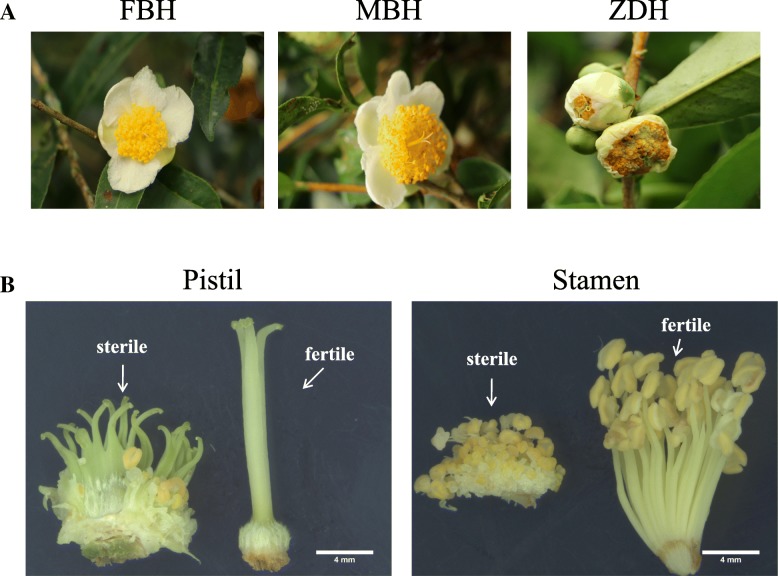
Morphological characteristics of the tea flower. **a** The morphologies of FBH, MBH and ZDH. **b** The morphologies of the fertile and sterile floral organ

The floral organ of the tea plant consists of a complete bisexual flower composed of a thalamus, calyxes, petals, stamens, and a pistil (Fig. 1a). In our study, the sterile flower buds were smaller than the male and female parent flower. Petals were improperly unfolding during the developmental process until flower buds abscission (Fig. 1a). The filaments were shorter than those of fertile flowers, and the anthers contained no pollen (Fig. 1b). In addition, sterile flowers contained two to four imperfect pistils, which were split into two to five smaller stigma (Fig. 1b).

### Transcriptome profiling of the male/female parent and the hybrid bud

We used the Illumina HiSeq 2000 platform to create three cDNA libraries, FBH, MBH, and ZDH. As a result, a total of 64.2, 60.6 and 74.6 million clean reads were generated. The quality score (Q30) percentage was above 92%, with the GC content of each clean data above 43%. The ratio of mapped reads was 73.64, 75.27 and 71.43%, respectively (Table 2). Afterwards, Trinity was used in splicing for clean reads. A total of 268,289 transcripts were obtained, and the longest transcript for each gene was selected as the unigene, of which 173,248 were screened for differential expression analysis (DEGs).

**Table 2:** Summary Dataset of Transcriptome Assembly

	male parent (FBH)	female parent (MBH)	sterile flowers (ZDH)
Clean reads	64,267,724	60,674,496	74,575,474
GC content	44.00%	44.17%	43.67%
Q30	92.41%	92.09%	92.53%
Mapped reads ratio	73.64%	75.27%	71.43%

### DEGs and functional characterization

**Fig. 2:**
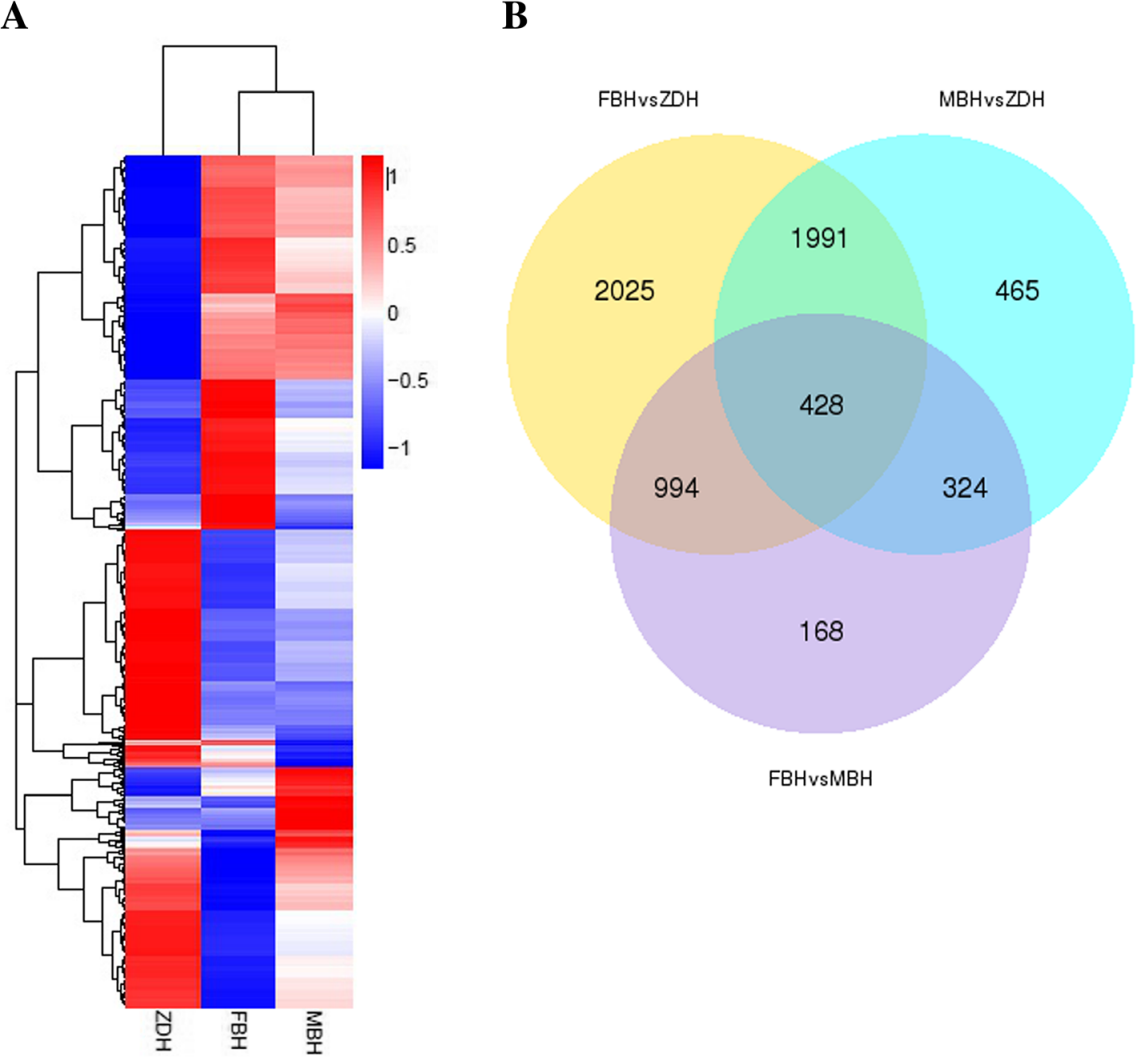
Cluster analysis of DEGs. **a** Heat map showing expression of the DEGs. High expression genes appear red, while low expression genes appear blue on the heat map. **b** The Venn diagram showing the number of DEGs between FBHvsZDH, MBHvsZDH, FBHvsMBH

A total of 6395 DEGs were detected in FBH, MBH and ZDH. 1914 DEGs were screened out between FBH and MBH. Between ZDH and FBH, the number of DEGs was 5438, whereas it was 3208 between ZDH and MBH. 1991 DEGs were screened out in ZDH, the expression quantity was different with FBH and MBH (Fig. 2).

**Fig. 3:**
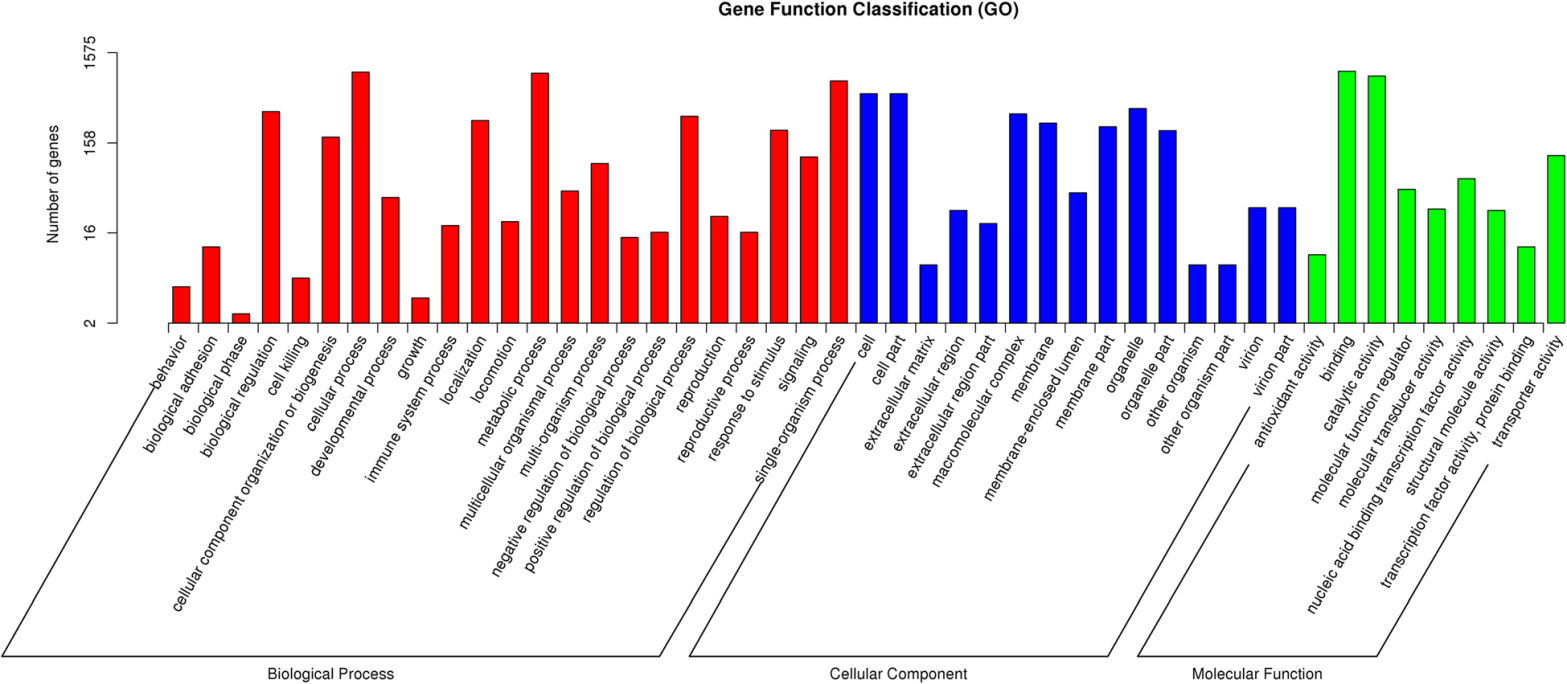
GO classification of DEGs. The red indicates biological processes, the blue indicates cellular components, and the green indicates molecular functions

The unique DEGs were characterized using the GO and KEGG databases. The GO enrichment analysis categorized 1991 unigenes, which contained 47 subcategories of biological processes, cellular components, and molecular functions. For the biological process, “biological regulation”, “cellular process”, “metabolic process”, and “single organism process” were the most representative groups. In the subcategory of cellular component, “cell”, “cell part”, “macromolecular complex”, and “organelle” were the predominant groups. For the molecular function, “binding”, and “catalytic activity” were the most common. Moreover, “reproduction”, “reproductive process”, and “growth” were enriched in 24, 16, and 3 unigenes, respectively (Fig. 3).

**Fig. 4:**
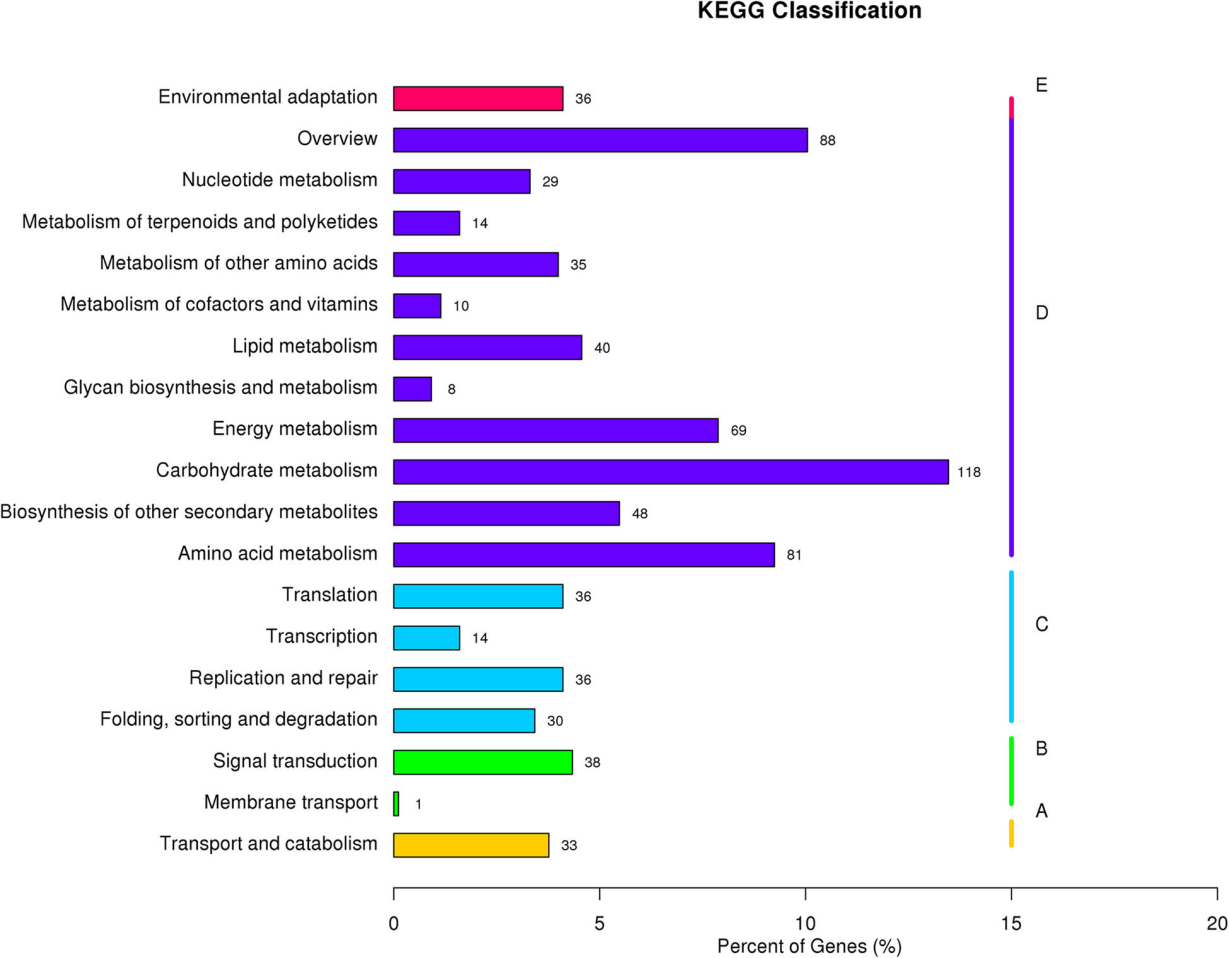
KEGG pathway classification of DEGs. **a** cellular processes; **b** environmental information processes; **c** generic information processes; **d** metabolism; **e** organismal systems

The KEGG analysis revealed that 764 unigenes were mapped into KEGG, containing cellular processes, environmental information processes, generic information processes, metabolism, and organismal systems. The main KEGG pathways included “Carbon metabolism”, “Biosynthesis of amino acids”, “Starch and sucrose metabolism”, “Plant hormone signal transduction”, “Plant pathogen interaction”, and “Glycolysis/Gluconeogenesis” (Fig. 4).

### Identification of auxin-related genes involved in DEGs

Auxin is essential for flower organ development [18]. Among DEGs, the auxin biosynthesis gene *YUCCA* related to floral organ formation was down-regulated. The auxin flux-related *PIN* homolog gene related to gynoecium formation was down-regulated. In addition, we identified ten auxin response factors ARF (Cluster-23,036.113917, Cluster-23,036.14480, Cluster-23,036.14481, Cluster-23,036.85241, Cluster-23,036.27862, Cluster-23,036.29441, Cluster-23,036.70956, Cluster-23,036.66525, Cluster-23,036.54073 and Cluster-23,036.87364) related to flower maturation was up-regulated (Table 3).

**Table 3:** DEGs Related to IAA Signal Transduction

Gene ID	KO name	Annotation	Log_2_ ratio	
			FBH vs ZDH	MBH vs ZDH
Cluster-23,036.78079	YUCCA	indole-3-pyruvate monooxygenase YUCCA10	2.50	2.57
Cluster-23,036.68881	JAR1	Indole-3-acetic acid-amido synthetase GH3.5	4.81	4.59
Cluster-23,036.78790	ILR1	IAA-amino acid hydrolase ILR1-like 6	2.65	2.00
Cluster-23,036.63096	ILR1	IAA-amino acid hydrolase ILR1-like 4	4.29	3.59
Cluster-23,036.77706	PIN	auxin efflux carrier protein	3.34	2.87
Cluster-23,036.72193	AUX1/LAX	auxin transporter-like protein 4	2.45	2.44
Cluster-23,036.74178	AUX1/LAX	auxin transporter-like protein 3	2.99	2.69
Cluster-23,036.53622	Aux/IAA	Auxin-responsive protein IAA7	6.17	5.43
Cluster-23,036.68884	Aux/IAA	auxin-induced protein 22D-like	3.48	3.33
Cluster-23,036.74950	Aux/IAA	auxin-induced protein 22D-like	5.43	5.34
Cluster-23,036.113917	ARF	auxin response factor 9	−4.76	−4.09
Cluster-23,036.14480	ARF	auxin response factor 5	−7.27	− 4.02
Cluster-23,036.14481	ARF	auxin response factor 5	−6.00	−4.96
Cluster-23,036.85241	ARF	auxin response factor 4	−2.60	− 2.02
Cluster-23,036.27862	ARF	auxin response factor 28	−3.23	−2.24
Cluster-23,036.29441	ARF	auxin response factor 28	−1.90	−1.09
Cluster-23,036.70956	ARF	auxin response factor 2	−1.41	− 1.11
Cluster-23,036.66525	ARF	auxin response factor 19-like	−1.38	−1.26
Cluster-23,036.54073	ARF	auxin response factor 19-like	−1.68	− 1.49
Cluster-23,036.87364	ARF	auxin response factor 1 isoform X1	−1.98	−1.77

### Identification of flower development-related genes involved in DEGs

Abnormal flower development is the key factor responsible for the sterility of the tea plant. The formation of flowers is a key step in the plant life cycle, which is a complex process [19]. Each stage of the process is regulated by flower development-related genes, specifically the ABC floral organ-identity genes [20]. We identified the A-class genes *AP1* and *AP2* were up-regulated.In addition, flower development-related genes, such as floral organ formation *SPL* homolog genes (Cluster-23,036.89600, Cluster-23,036.17164, Cluster-23,036.96383, Cluster-23,036.48034 and Cluster-23,036.89141) were up-regulated (Table 4).

**Table 4:** DEGs Related to Flower Development

Gene ID	KO name	Annotation	Log_2_ ratio	
			FBH vs ZDH	MBH vs ZDH
Cluster-23,036.89600	SPL8	squamosa promoter-binding-like protein 8	−3.52	−2.36
Cluster-23,036.17164	SPL6	squamosa promoter-binding-like protein 6	−2.32	−2.00
Cluster-23,036.96383	SPL3	squamosa promoter-binding-like protein 3	−4.85	−3.30
Cluster-23,036.48034	SPL12	squamosa promoter-binding-like protein 12	−1.68	−2.86
Cluster-23,036.89141	SPL9	squamosa promoter-binding-like protein 9	−2.75	−3.19
Cluster-23,036.69978	AP1	MADS-box transcription factor APETALA1-like	−1.92	−1.36
Cluster-23,036.57048	AP2	AP2-like ethylene-responsive transcription factor RAP2–7	−2.32	− 2.26
Cluster-23,036.116884	AP2	AP2-like ethylene-responsive transcription factor ANT	−8.76	−7.94
Cluster-23,036.90254	AGO5	protein argonaute 5	−3.32	−4.08
Cluster-23,036.85203	AGO4	protein argonaute 4	−2.48	−1.69
Cluster-23,036.99883	AGO2	protein argonaute 2	−1.61	−7.52
Cluster-23,036.95433	AGO10	protein argonaute 10	−3.76	−2.27

### Identification of energy transfer-related genes in DEGs

Energy transfer is an important process of plant growth and development. It is the foundation of the plant’s life [21]. We identified that some genes involved in energy transfer were differentially expressed, including ABC transporter B family member 1 (*ABCB1*), six transporting ATPase-related genes (*ATPeF0D*, *ATPeF0O*, *ATPeF1B*, *ATPeV0A*, *ATPeV1C*, and *ATPeV1B*), and five solute carrier-related genes (*SLC2A8*, *SLC15A3*, *SLC25A11*, *SLC32A*, and *SLC35B3*) (Table 5). A total of 12 genes were down-regulated. In particular, transporting ATPase subunit beta (*ATPeF1B*), solute carrier family 32 (*SLC32A*) and solute carrier family 35 (*SLC35B3*) were down-regulated 65.7, 60.9 and 54.9-fold, respectively, in ZDH compared with FBH. The expression levels of *ATPeF1B*, *SLC32A* and *SLC35B3* were also down-regulated 96.8, 34.7 and 65.4-fold, respectively, in ZDH compared with MBH.

**Table 5:** DEGs Related to Energy Transfer

Gene ID	KO name	Annotation	Log_2_ ratio	
			FBH vs ZDH	MBH vs ZDH
Cluster-23,036.67567	ABCB1	ABC transporter B family member 1	1.86	1.56
Cluster-23,036.73251	ATPeF0D	transporting ATPase subunit d	1.82	1.71
Cluster-23,036.68425	ATPeF0O	transporting ATPase subunit O	1.85	1.65
Cluster-23,036.67744	ATPeF1B	transporting ATPase subunit beta	6.03	6.58
Cluster-23,036.71789	ATPeV0A	transporting ATPase subunit a	1.92	1.46
Cluster-23,036.61724	ATPeV1C	transporting ATPase subunit C	1.77	1.37
Cluster-23,036.73958	ATPeV1B	transporting ATPase subunit B	1.49	1.21
Cluster-23,036.75094	SLC2A8	solute carrier family 2	2.86	2.31
Cluster-23,036.55114	SLC15A3	solute carrier family 15	1.87	1.49
Cluster-23,036.73478	SLC25A11	solute carrier family 25	2.48	1.87
Cluster-23,036.72951	SLC32A	solute carrier family 32	5.92	5.15
Cluster-23,036.49241	SLC35B3	solute carrier family 35	5.77	6.03

### Identification of other transcription factor genes in DEGs

We also identified some transcription factor genes. Among these genes, the expression levels of transcription factor AS1 (*AS1*), transcription factor CPC (*CPC*), and nuclear transcription factor Y (*NFYA*) were up-regulated significantly. Furthermore, MADS-box transcription factor (*K09264*), two transcription factor bHLH (*bHLH77* and *bHLH79*), three transcription factor MYB (*GAMYB*, *MYBP* and *MYB21*), transcription factor TGA6 (*TGA6*) and transcription factor GTE2 (*GTE2*) were down-regulated in ZDH (Table 6).

**Table 6:** DEGs Related to Transcription factors

Gene ID	KO name	Annotation	Log_2_ ratio	
			FBH vs ZDH	MBH vs ZDH
Cluster-23,036.105297	AS1	Transcription factor AS1	−2.43	−1.6
Cluster-23,036.65747	K09264	MADS-box transcription factor, plant	1.63	1.48
Cluster-23,036.17098	CPC	Transcription factor CPC	−3.25	−3.1
Cluster-23,036.14144	NFYA	nuclear transcription factor Y, alpha	−5.52	−6.14
Cluster-23,036.62850	bHLH77	Transcription factor bHLH77	2.68	2.92
Cluster-23,036.69683	bHLH79	Transcription factor bHLH79	6.53	5.59
Cluster-23,036.81661	GAMYB	Transcription factor GAMYB	2.46	2.42
Cluster-23,036.56315	GTE2	Transcription factor GTE2	5.82	6.92
Cluster-23,036.76553	MYBP	Transcription factor MYB108	7.47	6.01
Cluster-23,036.71827	MYB21	Transcription factor MYB21	4.64	4.09
Cluster-23,036.70389	TGA6	Transcription factor TGA6	5.48	5.2

### qPCR analysis of DEGs

**Fig. 5:**
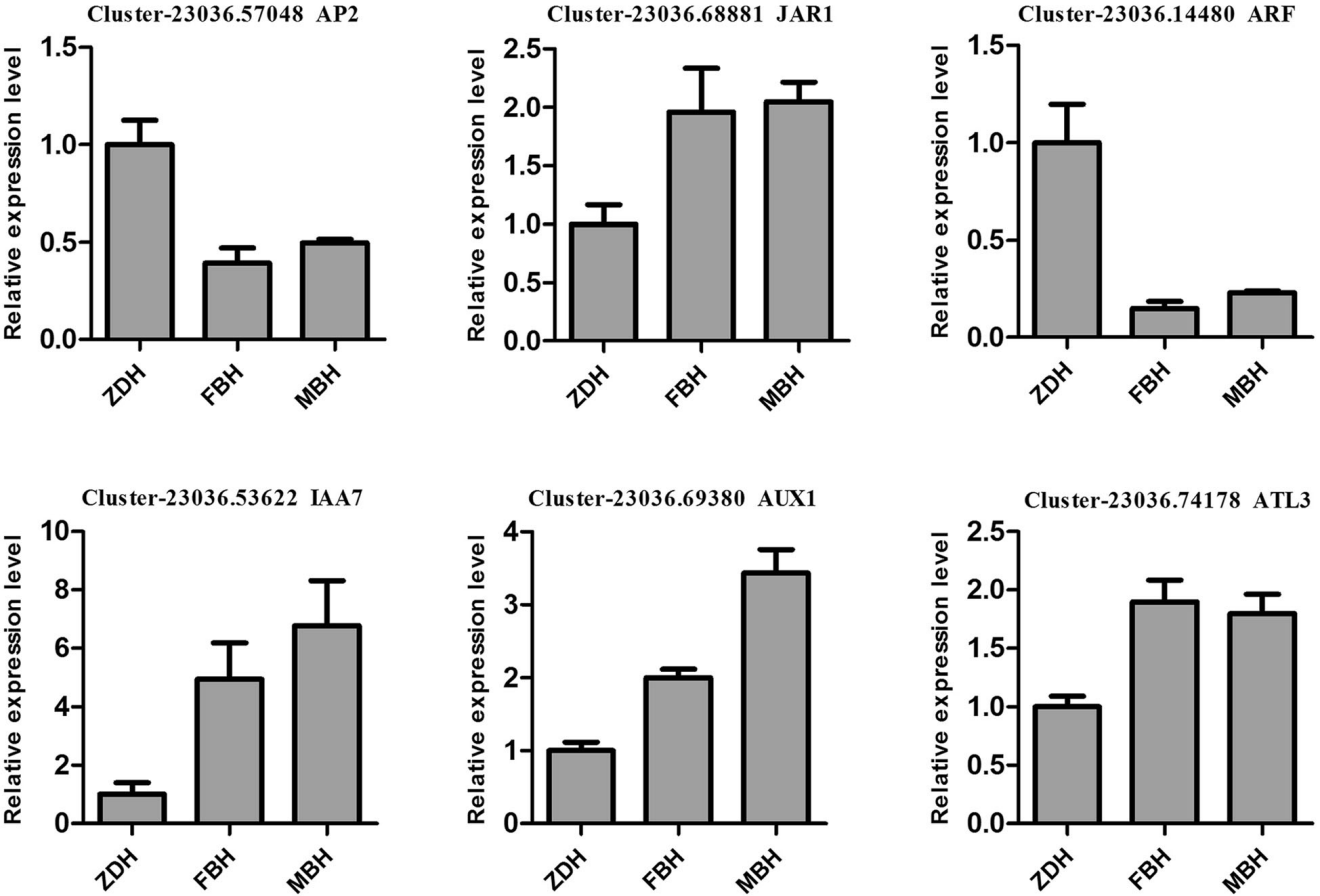
qPCR analysis of selected DEGs. Data represent the means ± SD, *n* = 3 independent experiments. ***p < 0.001 versus control

To confirm the transcriptome sequencing results, six DEGs were selected for qPCR analysis. Among these genes, Cluster-23,036.57048 (*AP2)* and Cluster-23,036.14480 (*ARF)* genes were up-regulated, and Cluster-23,036.68881 (*JAR1)*, Cluster-23,036.53622 (*IAA7)*, Cluster-23,036.69380 (*AUX1)* and Cluster-23,036.74178 (*ATL3)* genes were down-regulated (Fig. 5). qRT-PCR results showed a consistent expression tendency compared with the RNA-Seq. The result further confirmed the reliability and accuracy of the transcriptome sequencing.

## Discussion

Here, we used transcriptome sequencing to explore candidate genes associated with sterile floral buds in the tea plant. The method has been applied to the study of the genome for the tea plant [22–25]. A total of 1991 DEGs were screened out from the comparison among the three cDNA libraries (Fig. 2). The complexity of DEGs function was demonstrated by Go and KEGG analysis (Figs. 3 and 4). Based on the analysis, 452 metabolism-related pathways were identified (Fig. 4), suggesting that metabolism may be crucial for the sterility mechanism in the tea plant.

In general, the flavin monooxygenase (YUCCA) is involved in the tryptophan-dependent pathway of auxin biosynthesis [26], mutants of *YUCCA* present aberrant flower phenotypes, short stamen filaments, and thus sterility [27]. Our results show that the expression levels of *YUCCA* were down-regulated significantly (Table 3), the flower phenotype also appears as short stamen filaments (Fig. 1b). Thus, inferring that the low expression of *YUCCA* is a key factor affecting the biosynthesis of auxin leading to flower sterility. In addition, we also found that the expression of *SPL* homolog genes as an inhibiting factor of *YUCCA* was up-regulated in sterile flowers (Table 4). Previous studies showed that *SPL* represses *YUCCA* gene expression to regulate the development of lateral organs [28]. Our results suggest that *SPL* regulates the homeostasis of auxin by inhibiting *YUCCA*, resulting in flower sterility.

ARF play pivotal roles in the growth of inflorescences, stamens, anthers, and pistils. Mutations in *ARF6* and *ARF8* caused male and female infertility [29]. However, we found that the expression level of ten *ARF* homolog genes was up-regulated in sterile floral buds (Table 3). This is possibly due to the high expression of *ARF* inhibiting auxin signaling targets [30]. Moreover, ARF acts as a positive or negative regulator by binding to the auxin response element TGTCTC [31], and it is possible that the Aux/IAA inhibitor is able to inhibit transcription through interaction with ARF [32].

Interestingly, we found that the A-class of ABC floral organ-identity genes *AP1* and *AP2* were up-regulated in sterile floral buds (Table 4). It has been reported that the initial expression of *AP1* and *AP2* was restricted to the first and second whorls, and was inhibited in the third and fourth whorls of flower development [33]. Thus inferring that high expression of AP1 and AP2 represses flower development in first and second whorls. Detailed mechanisms will require further study.

As an important regulator of flower development, auxin is transported to each tissue through carriers and the AUX1/LAX [34]. We found that the expression levels of *AUX1/LAX* (Table 3) and twelve energy transfer-related genes (Table 5) were down-regulated, suggesting that the transport of auxin may be impeded. This may also be an important factor in the sterile floral buds of the tea plant.

In summary, the present work provides four key factors for the development of sterile floral buds in the tea plant. We thus present a theoretical basis for further study of mechanisms by which sterile floral buds are produced.

## Abbreviations

DEGs: Differentially expressed genes
FBH: Foxiang2
GO: Gene Ontology
KEGG: Kyoto Encyclopedia of Genes and Genomes
MBH: Fudingbaicha
ZDH: Hybrid sterile flowers
